# Long-Term Effects of Temporal Lobe Epilepsy on Local Neural Networks: A Graph Theoretical Analysis of Corticography Recordings

**DOI:** 10.1371/journal.pone.0008081

**Published:** 2009-11-26

**Authors:** Edwin van Dellen, Linda Douw, Johannes C. Baayen, Jan J. Heimans, Sophie C. Ponten, W. Peter Vandertop, Demetrios N. Velis, Cornelis J. Stam, Jaap C. Reijneveld

**Affiliations:** 1 Department of Neurology, VU University Medical Center, Amsterdam, The Netherlands; 2 Department of Neurosurgery, Neurosurgical Center Amsterdam, VU University Medical Center, Amsterdam, The Netherlands; 3 Department of Clinical Neurophysiology and Epilepsy Monitoring Unit, Dutch Epilepsy Clinics Foundation, Heemstede, The Netherlands; 4 Department of Clinical Neurophysiology, VU University Medical Center, Amsterdam, The Netherlands; 5 Department of Neurology, Academic Medical Center, Amsterdam, The Netherlands; Indiana University, United States of America

## Abstract

**Purpose:**

Pharmaco-resistant temporal lobe epilepsy (TLE) is often treated with surgical intervention at some point. As epilepsy surgery is considered a last resort by most physicians, a long history of epileptic seizures prior to surgery is not uncommon. Little is known about the effects of ongoing TLE on neural functioning. A better understanding of these effects might influence the moment of surgical intervention. Functional connectivity (interaction between spatially distributed brain areas) and network structure (integration and segregation of information processing) are thought to be essential for optimal brain functioning. We report on the impact of TLE duration on temporal lobe functional connectivity and network characteristics.

**Methods:**

Functional connectivity of the temporal lobe at the time of surgery was assessed by means of interictal electrocorticography (ECoG) recordings of 27 TLE patients by using the phase lag index (PLI). Graphs (abstract network representations) were reconstructed from the PLI matrix and characterized by the clustering coefficient C (local clustering), the path length L (overall network interconnectedness), and the “small world index” S (network configuration).

**Results:**

Functional connectivity (average PLI), clustering coefficients, and the small world index were negatively correlated with TLE duration in the broad frequency band (0.5–48 Hz).

**Discussion:**

Temporal lobe functional connectivity is lower in patients with longer TLE history, and longer TLE duration is correlated with more random network configuration. Our findings suggest that the neural networks of TLE patients become more pathological over time, possibly due to temporal lobe changes associated with long-standing lesional epilepsy.

## Introduction

Epilepsy surgery is often a more effective way to treat temporal lobe epilepsy (TLE) than anti-epileptic drugs (AEDs) [1], [2], but it is considered a last resort by many physicians. This reluctance can be explained by a failure of the surgical procedure to alleviate epileptic seizures in a considerable number of cases and by the risk of postoperative cognitive and visual deficits. Another reason for a delay in surgery is that TLE is often characterized by periods of remission with relative seizure freedom [3]. As a result, a history of epileptic seizures of 10 to 20 years prior to surgical intervention is not uncommon [4].

As most patients in whom surgery is considered suffer from TLE for a period of at least several years, it is important to understand how this ongoing disease interferes with brain functioning. If a prolonged disease course has a negative impact on brain functioning in TLE patients, this would support the importance of early surgical intervention. However, relatively little is known about the natural history of TLE. It is often preceded by an initial precipitating injury, of which a complex febrile seizure is the most common [5]. Hereafter, a latent period tends to occur, followed by recurrent, spontaneous seizures, indicating that TLE might be a progressive disease [6]. When looking at structural damage related to epileptic seizures, progressive volume loss of the hippocampus, amygdala and the entorhinal cortex has been described as a consequence of ongoing TLE [4]. A correlation between the number of brain structures with epileptogenic characteristics and epilepsy duration has been described in TLE patients [7]. It is not known how functional neural networks change during disease progression, and what the impact of changes is on seizure initiation and propagation. However, a contralateral increase of functional connectivity for TLE patients has been described [8].

**Figure 1:**
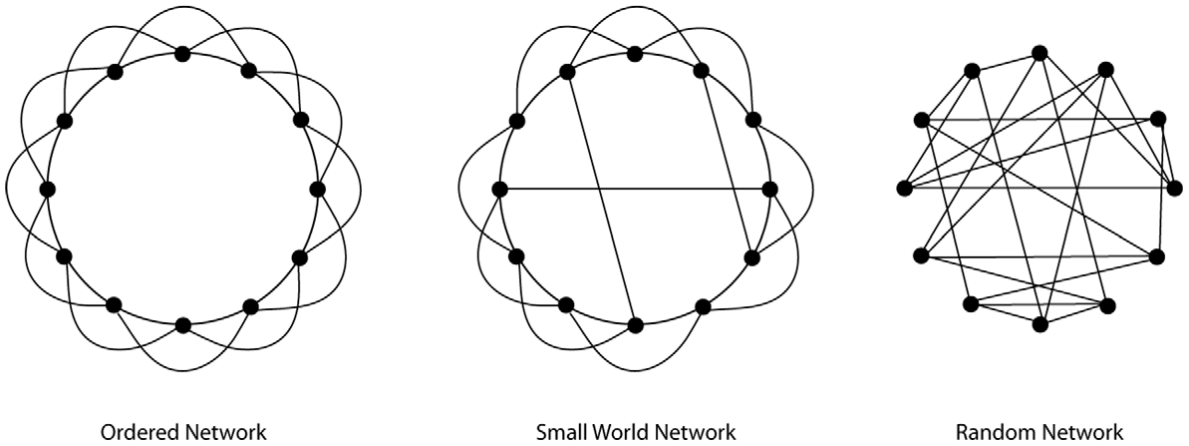
Three network types. Three network types based on the model of Watts and Strogatz, 1998. In an ordered network the points are connected to their nearest neighbours (left) but there are no long-distance connections. In a random network (right), there is no local clustering. In a small-world network (middle), some local connections are rewired to long distance connections, resulting in high clustering combined with a short path length.

In modern neuroscience, the brain is increasingly seen as a complex network of dynamical systems with interactions between local and further remote brain regions. A way to explore the interactions between brain regions is to look at functional interactions, also called functional connectivity. Functional connectivity refers to the statistical interdependencies that exist between neurophysiological time series [9]. Electrocorticography [10] and depth electrode [11]–[13] studies have shown that network synchronization in the temporal lobe increases during a seizure when compared to the interictal and postictal states. Several studies indicate that a predisposing state exists prior to a seizure, which is characterized by desynchronization or hypersynchronization in different surrounding brain areas [14]–[16]. The changing synchronization patterns may be explained by the impact of brain disease on the spatial network configuration of the brain. These changes can be studied using a ‘graph’ theory approach [9]. A ‘small world’ network is thought to be the optimal network configuration for brain functioning [17]. In such a small world network, local integration is high, while the overall integrity of the network is also maintained (see figure 1). Social networks, the Internet, and the healthy brain are examples of networks that show these characteristic small world features [9]. It is suggested that changes in network characteristics occur in epilepsy patients, leading to a pathological, more random structure in the interictal state, which is temporarily reversed during a seizure [10]–[12], [18], [19]. These changes in configuration may lead to disturbed higher brain functions and further lower the brain's threshold for epileptic seizures (for a review see [20]).

A better understanding of the effects of ongoing TLE on neural functioning might influence the timing of surgical intervention. Functional connectivity and neural network analysis have shown to be a promising tool in studying epilepsy and brain tumor patients. Changes in network characteristics and functional connectivity have been associated with both epilepsy and brain lesions [10], [15], [19], [21]. We therefore consider that changes in functional connectivity and network configuration may be a marker of possible progression of TLE. We hypothesize that functional interactions in the brain are correlated to temporal lobe epilepsy duration. We expect to demonstrate a correlation between TLE duration and changes in network characteristics of the temporal lobe. We expect less functional connectivity and a more random configuration of the temporal neural networks as TLE duration increases.

## Methods

### Patients and Data Selection

All pharmaco-resistant TLE patients who underwent temporal lobe surgery and intraoperative ECoG recordings at the VU University Medical Center in Amsterdam between October 2003 and September 2005 were eligible for inclusion in this study. The VU University Medical Center is a tertiary referral centre for epilepsy surgery as part of the nationwide Dutch Collaborative Epilepsy Surgery Program.

**Figure 2:**
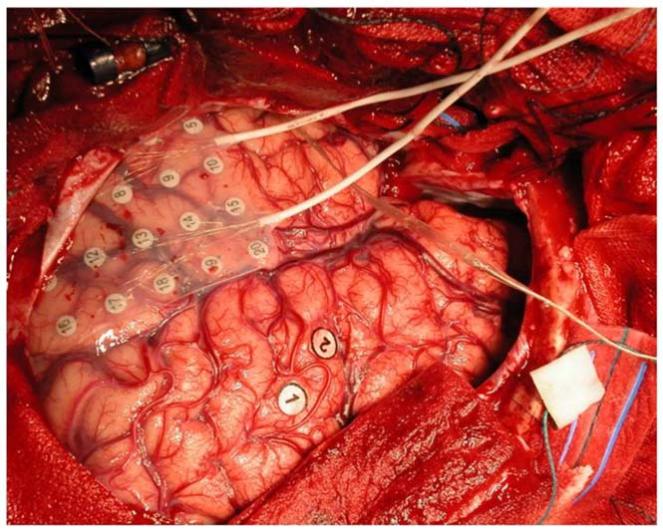
Intra-cranial corticography recording. Picture of a grid, placed directly on the cortex for intra-cranial recording.

Intra-operative neocortical registrations were performed using 4×5 subdural electrode grids (interelectrode distance 1 cm; see figure 2) and recorded with a Brainlab ® digital EEG system (OSG, Rumst, Belgium). Sample frequency was 500 Hz. The grid was placed directly on the lateral temporal cortex, covering T1-T3. The grid position was documented during the procedure on schematic representations of the brain. For every patient, five artefact-free epochs of 4096 samples (8,19 s) were selected off-line by careful visual inspection (ED, CJS). All selected epochs were recorded while patients were in an interictal state (as determined by the ECoG recording) and before resection of the lesion had taken place.

Patients were sedated with propofol during surgery. Due to the retrospective nature of this study, there was no standardized protocol to control the amount of anaesthesia while recording ECoG. As the level of sedation might influence the ECoG, the burst suppression ratio (BSR) of the recordings was calculated and used as a covariate in the analysis (See Figure S1 for an example of used ECoG data). The BSR is the sum of all the regions showing at least 0.5 seconds of suppression, divided by the total length of the epochs (40.95 s). Suppression was defined as a region with very low ECoG activity, using 5 microV as threshold value [22].

### Ethics Statement

The data were obtained for clinical purposes with patient consent for all performed procedures. As a retrospective study this work was exempt from ethical approval. Data were analyzed anonymously. All clinical investigations were conducted according to the Declaration of Helsinki.

### Functional Connectivity

**Figure 3:**
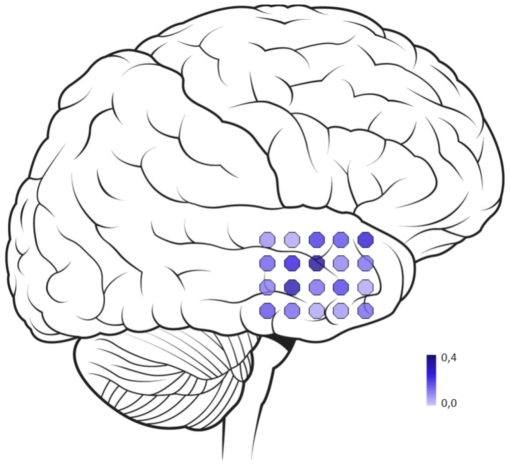
Visualisation of the functional connectivity pattern over the temporal lobe. A grid of 4x5 electrodes is placed directly on the temporal lobe and the registered area is schematically documented. 5 Epochs of representative ECoG recordings are selected per patient. The PLI values are calculated based on these recordings, resulting in a matrix with synchronization values for all possible electrode combinations. The figure shows a mean PLI value for each grid electrode based on this synchronization matrix, representing the average synchronization with the other channels.

Selected epochs were converted to ASCII files for further analysis with DIGEEGXP software, developed in our department (CJS). Five selected epochs per patient were filtered in the broad frequency band (0,5–48 Hz). The phase lag index (PLI) was used to calculate functional connectivity of the selected epochs [23]. The PLI measures the asymmetry of the distribution of instantaneous phase differences between different EEG signals. The PLI rules out volume conduction as a confounding factor, since the presence of a consistent, nonzero phase lag between two time series cannot be explained by volume conduction alone. The PLI is therefore less effected by common sources, while performing at least as well as the synchronization likelihood (SL) [24] in detecting true synchronization. The PLI ranges between 0 and 1. A PLI of more than 0 indicates phase locking to a certain extent, whereas a PLI value of 0 indicates no coupling or coupling with a phase difference centered around 0±π radians. For a detailed description of PLI calculation, see Stam [23]. The PLI was calculated between all channels, resulting in a 20 by 20 channel matrix. The overall (whole grid) PLI was computed by averaging all PLI values. To visualize functional connectivity, we projected the PLI scores per channel over a schematic grid representation, showing the average synchronization of the channel with the other channels of the grid (see figure 3).

### Graph Analysis

We hypothesize that network topology, and not just average strength of synchronization as indicated by PLI, is correlated with epilepsy duration in our study population. A graph is a basic topographical representation of a network that consists of nodes (‘vertices’) and connections between these nodes (‘edges’) [17]. Graphs are characterized by a clustering coefficient C and a characteristic path length L. The clustering coefficient C, which is the likelihood that neighbors of a vertex will also be connected, is a measure for the tendency of network elements to form local clusters. The characteristic path length L is the average of the shortest distance between pairs of vertices counted as a number of edges. The path length L indicates how well network elements are integrated or interconnected [17].

To compute the clustering coefficient C and the characteristic path length L from the PLI of the ECoG data, we used the methods described by [25]. The first step in applying graph theoretical analysis to synchronization matrices is to convert the N×N PLI matrix into a binary graph, with N as the number of channels used. A binary graph is a network that consists of vertices and edges (undirected connections between elements). The PLI matrix can be converted to an unweighted graph by considering a threshold T. If the PLI between a pair of channels i and j exceeds T, an edge is said to exist between i and j; otherwise no edge exists between i and j.

Differences between the patients in average PLI can influence C and L when it is computed for a threshold T, because a lower PLI trivially results in less edges. To control for the influence of the number of edges, we applied several fixed values of k (k = 3, k = 4, k = 5), where k denotes the average number of edges per vertex. By using this method, graphs in both groups are guaranteed to have the same number of edges. We followed the suggestion of [17] for the minimal k value for a network with size N (here 20), such that a random network generated by using this k will still be fully connected: N≥k≥Ln(N). In the current study, this was true for k≥3. K values between 3 and 5 have been used in previous studies with a similar network size [25], [26].

Once the PLI matrix has been converted to a graph, the next step is to characterize the graph in terms of its clustering coefficient C and characteristic path length L. The path length L was calculated as ‘harmonic mean’ distance between pairs as described by [27], making it possible to deal with vertices that are not connected. The values of C and L for every k were compared to 1000 random surrogate matrices, generated as described by [28], by calculating the ratio between the C and L of the patient and the surrogate data (referred to as C-s and L-s). To analyze the small world characteristics of the network we used the measure of small world index S [29], which is defined as S  =  (C/C-s)/(L/L-s). A network can be defined as a small world network if C/<C-s>≫ 1 and L/<L-s> ∼1, which means that any value of S greater than 1 is account for small world network.

### Statistical Analysis

All statistical analyses were performed using SPSS15.0 for Windows (SPSS Inc., Chicago, USA).

Possible correlations between age, age of epilepsy onset and epilepsy duration were analyzed by means of Pearson's correlation coefficients.

Analysis of the correlation between epilepsy duration and the type of lesion or type of epilepsy was done using independent *t*-tests (MTS versus tumor; partial versus generalized seizures) and one-way ANOVA (differentiated for multiple types of lesions and seizures).

In order to analyze correlations with seizure frequency, BSR, PLI and network variables, non-parametric tests were used, because these variables were not normally distributed. Correlations between PLI and network characteristics and epilepsy duration, age, age of epilepsy onset, seizure frequency, and BSR, were analyzed by means of Kendall's Tau correlation coefficients. Possible effects of gender, seizure type (partial or generalized), nature of the lesion (tumor, vascular or mesial temporal sclerosis), and AEDs (monotherapy or polytherapy) on PLI and network characteristics were analysed by means of Mann-Whitney nonparametric *U*-tests.

Since multiple correlations were analyzed, a Bonferroni correction was performed.

## Results

### Patient Characteristics

The files of a total of 27 TLE patients (mean age 40 years, 41% male) were analyzed (for patient characteristics, see table 1 and 2). Surgical outcome after one year was documented according to the modified Engel scale [30]. Three patients showed artefacts in the ECoG recordings, which occurred in one channel (one patient) or two channels (two patients). These artefacts were caused by bad contacts between the grid and the temporal brain surface. The artefact channels were excluded from further analyses in these patients.

**Table 1:** Clinical data.

Patient	Gender	Age	History of epilepsy (yrs)	Seizure type	Path	Outcome (one year)
1	F	36	22	Partial simple and complex	DNET	IA
2	M	41	5	Partial simple and complex	MTS	IB
3	F	35	3	Sec. generalized; partial complex	LGG	IA
4	M	49	42	Partial complex	MTS	IA
5	M	41	0	Partial simple and complex	LGG	IB
6	M	21	3	Generalized; partial complex	DNET	IA
7	M	18	12	Partial complex	MTS	ID
8	M	57	3	Partial complex	AVM	IA
9	F	68	32	Partial complex	MTS	IA
10	F	48	15	Partial complex	Unknown	IA
11	F	17	16	Generalized	MTS	IA*
12	M	38	36	Partial complex	MTS	IA
13	F	20	3	Partial simple	MTS	IA
14	F	37	30	Partial complex	MTS	IA
15	F	35	25	Generalized; partial complex	LGG	IA
16	F	52	50	Generalized; partial complex	MTS	IA
17	F	25	24	Sec. generalized; partial simple and complex	MTS	IIA
18	M	67	20	Generalized; absence, partial complex	HAEM	IID
19	F	42	15	Generalized, partial complex, partial simple	LGG	IA
20	M	50	4	Absence	HAEM	IA
21	M	33	21	(Sec.) generalized; partial complex	MTS	IIB
22	F	50	25	Sec. generalized; partial complex	MTS	IIB
23	F	41	4	Partial simple and complex	HAEM	IA
24	F	28	8	Sec. generalized; partial simple	MTS	IB
25	F	42	28	Generalized; partial complex	MTS	IID
26	M	41	25	Partial complex	Unknown	IB
27	F	43	15	Sec. generalized; partial simple and complex	MTS	IA

Abbreviations: F = female; M = Male; yrs = years; Path = pathology; DNET = Dysembryoplastic Neuroepithelial Tumor; MTS = Mesiotemporal sclerosis; LGG = Low-Grade Glioma; AVM = Arteriovenous malformation; HAEM = Cavernous Haemangioma; Unknown = tumor of unknown pathology; Outcome (one year) = Clinical outcome one year after surgery according to a standardized scale of epilepsy burden (Engel et al, 1993); IA = seizure free; IB = non-disabling partial seizures; IC = some disabling seizures; ID = generalized seizures only when AED is stopped; IIA = First seizure free, now rare seizures IIB = rare disabling seizures; IIC = More then rare disabling seizures since surgery; IID = nocturnal seizures only.

* = Data were only available at 6 months after surgery.

**Table 2:** Patient characteristics.

Category	Subcategory	Result (±SD)	Correlation with epilepsy duration
Age		40±13	.
Gender	Male	41%	.
	Female	59%	
Type of lesion	MTS	56%	.
	Tumour	37%	
	Unknown	7%	
History of epilepsy (years)		18±13	Not applicable
Seizure frequency (per year)		636±718	−0.378 (p = 0.009)
Age of onset (years)		22±16	−0.589 (p = 0.001)
Type of epilepsy	Partial	56%	.
	Generalized	44%	
Antiepileptic drug use	Monotherapy	33%	.
	Polytherapy	67%	
BSR		0.15±0.13	−0.307 (p = 0.030)*

Several characteristics of the patient population. Seizure frequency is reported as an extrapolation of the patient's medical record, e.g. one seizure per week was reported as 52 seizures per year. Correlations with epilepsy duration were given if significant.

* = The correlation between BSR and epilepsy duration was no longer significant after Bonferroni correction.

Four correlation coefficients were calculated regarding epilepsy duration and its correlation with other patient characteristics (age, age of epilepsy onset, BSR, seizure frequency). A Bonferroni correction for multiple analysis was performed, determining that significant correlations were found only when p<0.013 level was reached.

### Functional Connectivity

**Figure 4:**
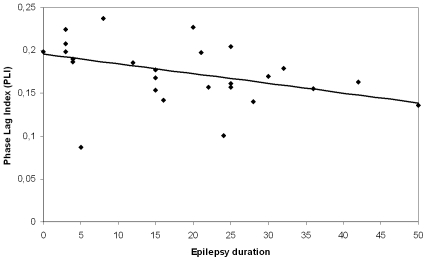
Correlation between epilepsy duration and functional connectivity. The Phase Lag Index (PLI) value is given as a function of epilepsy duration of each patient, showing a decreased PLI with longer epilepsy duration.

The overall PLI was significantly correlated with the history of epilepsy: PLI was lower when epilepsy duration was longer (Kendall's Tau = −0.389; p = 0.005) (see Figure 4). Correlations with PLI are shown in table 3. We found a significant correlation between the PLI and BSR, indicating that a higher level of sedation was correlated with increased PLI. Also, higher PLI was correlated with a higher age of onset. No correlation was found between seizure frequency and PLI. The significance level was set at p<0.013 for correlations of patient characteristics (epilepsy duration, age of onset, BSR, seizure frequency) with PLI, because four correlations were calculated. The correlations between epilepsy duration and BSR with PLI, remained significant after the Bonferroni correction.

**Table 3:** Correlations with functional connectivity and network characteristics.

Category	PLI	C/Cs (k = 3)	L/Ls (k = 3)	S (k = 3)
Age of onset	0.390 (p = 0.005)	.	.	.
History of epilepsy	−0.389 (p = 0.005)	−0.346 (p = 0.013)	.	−0.360 (p = 0.009)
Seizure frequency	.	0.376 (p = 0.009)	.	0.412 (p = 0.004)
Antiepileptic drug use	U = 17 (p<0.001)	.	.	.
BSR	0.551 (p<0.001)	.	.	.

All significant correlations with PLI and network characteristics. Antiepileptic drug use is shown as a Mann-Whitney U test value, reflecting a significantly lower PLI in monotherapy versus polytherapy. All other values represent correlation coefficients with corresponding p-values.

Group comparisons were made to analyze possible correlations between gender, lesion type or single versus multiple AED use and PLI. Mann-Whitney U Test showed a significantly lower PLI in patients using multiple AEDs (M = 0.159) when compared to single AED use (M = 0.207). No correlation was found between lesion type or gender and PLI. The PLI difference for single versus multiple AED use remained significant after Bonferroni correction (p<0.025 as two comparisons were calculated).

### Graph Analysis

**Figure 5:**
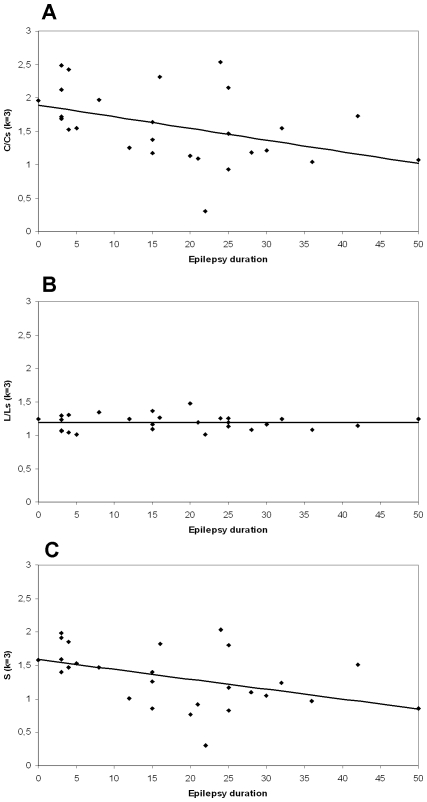
Correlation between epilepsy duration and network characteristics. The clustering coefficient C and average shortest path length L were calculated for each patient and compared to 1000 random networks, resulting in a ratio C/Cs and L/Ls. Network characteristics were plotted into a graph as a function of epilepsy duration, showing a decrease of C/Cs (5A) and no change of L/Ls (5B), resulting in a decrease of the small world index S (5C). The shown results represent the network characteristics for an average number of connections k = 3.

**Figure 6:**
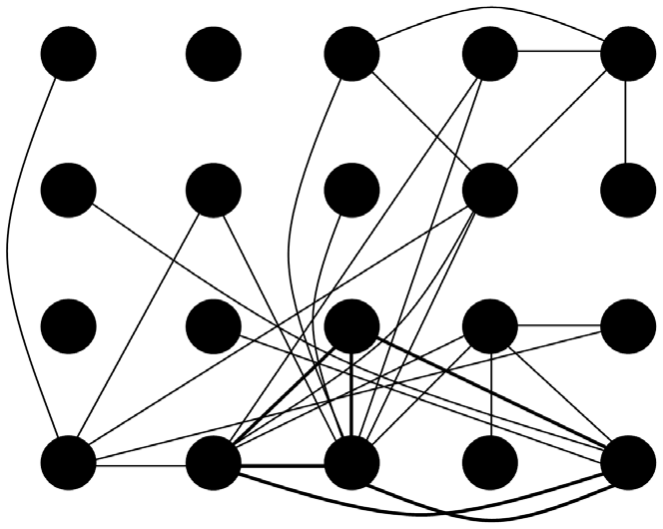
Network representation on the ECoG grid. Network representation of patient 13 for an average number of connections k = 3. The characteristics of this network are: C/Cs = 2.12, L/Ls = 1.07 and S = 1.98. Note that some edges are printed bold. These edges represent connections between three so-called ‘hub’ vertices (vertices with a relative high number of connections), which increase the small world properties of the network in two ways. Firstly, the edges mark a local cluster, which increases the network characteristic C/Cs. Secondly, this particular cluster also increases the global integration of the network as almost all connected vertices in the network can be reached through these hubs, resulting in a lower L/Ls.

The clustering coefficient C and small world index S were correlated with history of epilepsy for an average degree k = 3 (Kendall's Tau (C) = −0.346; p = 0.013 and Kendall's tau (S) = −0.360; p = 0.009 respectively) (see figure 5). The correlation of epilepsy duration with the small world index S remained significant after Bonferroni correction, determining correlations to be significant when p<0.013 was reached. The clustering coefficient C showed a correlation with epilepsy duration at a significance level of p = 0.013, which was the same as the cut-off point for significance after Bonferroni correction. An example of a network distribution over the ECoG grid is shown in figure 6.

When using an average degree k = 4, clustering coefficient and small world index tended to be correlated to epilepsy duration (Kendall's tau (C) = −0.236; p = 0.090 and Kendall's tau (S) = −0.226; p = 0.103 respectively), but did not reach the significance level (p<0.013). No group differences were found regarding graph analysis when using k = 5.

There were no significant correlations between gender, age of epilepsy onset, AED use, or BSR with respect to clustering coefficient C, path length L, and small world index S for the used values of k. However, a correlation was found between seizure frequency and clustering coefficient C and between seizure frequency and small world index S for k = 3: higher seizure frequency was associated with higher local clustering and a higher small world index of the network. These correlations were still found to be significant after Bonferroni correction (significant correlation when p<0.013). There were no correlations between seizure frequency and local clustering, path length, and small world index for k = 4 and k = 5.

Several interactions between patient characteristics were analyzed, as mentioned in the methods section (table 2). A significant negative correlation was found between epilepsy duration and age of epilepsy onset (Pearson correlation  = −0.589; p = 0.001), indicating that patients with a longer epilepsy duration were younger at epilepsy onset. We also found a negative correlation between epilepsy duration and seizure frequency (Kendall's Tau  = −0.378; p = 0.009). This indicates that patients with a longer history of epilepsy had a lower seizure frequency. Epilepsy duration tended to be negatively correlated with the BSR (Kendall's Tau  = −0.307; p = 0.030), indicating a lower sedation level during ECoG recordings in patients with a longer epilepsy duration. However, the significance level for this interaction was no longer reached after Bonferroni correction. No other interactions were found between the patient characteristics.

## Discussion

### Functional Connectivity

This study indicates that broad-band functional connectivity recorded intra-operatively over the temporal neocortex is lower in patients with a longer history of TLE. The association between pathology and lower functional connectivity is in line with other studies. Correlations between functional connectivity loss and pathological states such as epilepsy, brain tumors, and schizophrenia have been described previously [15], [21], [31]. Bartolomei and others found a correlation between duration of epilepsy and the number of cortical structures with epileptogenic characteristics by analyzing intracerebral EEG recordings of TLE patients [7]. A correlation has been reported between brain tumors and decreased broad-band functional connectivity [21]. It is hypothesized that pre-ictal hypersynchronized epileptogenic zones may be surrounded by isolating zones of hyposynchronization [15]. Ponten and others found that synchronization increases during a seizure in TLE patients [11]. Based on these studies, it is hypothesized that epileptogenic zones might be identified by their synchronization pattern. Synchronization analysis may therefore be useful as a method to functionally map the temporal cortex of TLE patients, hereby locating specific sites that participate in the initiation and propagation of seizures [32], [33]. Furthermore, analyzing synchronization patterns might be a way to locate epileptic foci in TLE patients who are selected for epilepsy surgery. Ortega and colleagues have suggested that seizures might arise from specific regions that have synchronization patterns which are highly differentiated from patterns in the rest of the temporal cortex [32], [34].

### Network Properties

This study shows that patients with a longer TLE duration have less small world network properties in the temporal cortex, suggesting that a less optimal functional network configuration occurs in the course of TLE. This study is the first to show an association between changes in neural network characteristics and the duration of TLE.

The correlation between increased network randomization and increased TLE duration is found to be most pronounced when using a high threshold for the number of edges (k = 3). The choice of a threshold is somewhat arbitrary, but also provides information about the connections that change over time. We speculate that in this study, more random networks in patients with a longer epilepsy duration reflect a randomization of the strongest local connections. This randomization results in a less optimal local network configuration, which might reflect a higher vulnerability for seizures.

Morgan and Soltesz [35] found that the incorporation of a small number of highly interconnected granule cell hubs into a rat dentate gyrus model greatly increases network activity. In their model, this resulted in a hyperexcitable, potentially seizure-prone circuit.

Several other model studies describe the potential importance of network randomization regarding the vulnerability to seizures. Netoff and co-workers found in a hippocampal slice model that seizures could be induced by changing the proportion of local versus long-distance connections [36]. As the neural network configuration was transformed into a more random network, seizure-like behavior was more likely to arise. Srinivas and others observed hippocampal rat neurons in vitro which were injured with an exposure to glutamate [37]. The neural network became hypersynchronous and fired bursts at high frequency after this injury, which they interpreted as induced epileptic activity. The network properties showed that the clustering coefficient decreased after injury: the network became more random as epileptic activity developed. Percha and others found that in a model of mesial TLE, epileptogenesis is characterized by structural changes in the neural network topology and axonal sprouting [18]. They showed in a two-dimensional model that an abrupt transition from an unordered local state to an ordered state of global coherence occurs when the network configuration changes from a small-world network to a more random network configuration. The authors speculated that a seizure arises as brain pathology causes the transition to a more random network beyond a critical point.

But what causes this randomization? The lesions in TLE patients result in cell loss, which might cause a reduction of the number of connected edges [38], as has been described in brain tumor patients [19]. Apart from cell loss, mossy fiber sprouting is a hallmark of seizure-induced sclerosis in TLE. Dyhrfjeld-Johnsen and others used a model with cell loss and sprouting to simulate network changes in TLE patients [39]. The value of L/L_random_ = 1,19 found in the Dyhrfjeld-Johnsen study at baseline (without a sclerotic process) was the same as the average path length found in our study. When looking at progression of sclerosis, we see that one of their simulation models, based on mossy fiber sprouting, also has a pattern similar to ours. In this model, L/L_random_ remains stable while C/C_random_ becomes more random with increasing sclerosis. Mossy fiber sprouting might therefore explain our findings of a more random network with increased epilepsy duration.

Previous clinical studies have already shown a correlation between dynamic functional network properties and TLE. Wendling and others found that the interaction of local interneuron connections in TLE patients is involved in the interictal to ictal state transition [14], [35]. Ponten and co-workers analyzed the network configuration of mesial TLE patients based on the synchronization likelihood [11]. They compared network topography before, during, and after a seizure. A more randomly organized network was present in the interictal state, which moved towards a more ordered configuration during seizures. Most changes occurred in the C/C-s ratio. Schindler and colleagues found a similar result in their analysis of EEG recordings of 100 epilepsy patients [12]. Their study showed an increase of C/C-s ratio and L/L-s ratio during seizure onset, and a decrease of these parameters during seizure end. Kramer and others also found a changing clustering coefficient and small world index at seizure onset [10]. Our study suggests that a long history of seizures (which induces shifts in network topology) may be related to functional changes in interictal local clustering and small world properties.

An unexplained phenomena in TLE is its course, which is often characterized by a seizure-free latency period after initial onset, eventually ended by recurrence of seizures. Because of this course, it is suggested that TLE should be seen as a progressive disease. Bernasconi and others found progressive volume loss of the hippocampus, the amygdala, and the entorhinal cortex in TLE patients, which is the structural manifestation of progression [4]. The possible emergence of an epileptogenic network during the silent period of TLE has been described [40]. There seems to be a randomness threshold, beyond which a network is prone to seizure-like behaviour. Neural networks of TLE patients may become more random during the latency period, allowing this threshold to be crossed and thus resulting in seizures recurrence [41], [42]. Our findings support the hypothesis of such a transition, changing the network configuration over time to a more random state which is more prone to seizures.

Furthermore, the recurrence of a single seizure might lower the threshold for new seizures to occur in TLE patients [6], [43]. From this perspective, it might be hypothesized that not only structural changes due to the lesion alter the functional network configuration, but the seizures do as well. We speculate that lesion and seizures affect the local neural networks in such a way that they become more prone to seizures, which might play an important role in the recurrence of epilepsy after a latency period in TLE patients.

Age of epilepsy onset, AED use, and level of anaesthesia during surgery must be considered as possible confounders in this study regarding functional connectivity, as these factors were correlated with both epilepsy duration and functional connectivity. However, the found correlation remained a significant result after Bonferroni correction, indicating that the result was robust and was not explained by multiple testing. Age of onset was not correlated with network characteristics (C, L, and S). Likewise, AED use and level of anaesthesia during surgery were related to synchronization, but not to network configuration measures. These findings show that network characteristics, although based on functional connectivity, measure different functional properties. Lesion volumes were not available and were therefore not included in our analysis.

In our study, we have found a correlation between higher seizure frequency and a higher clustering coefficient and small world index. We also found, however, a higher seizure frequency to be correlated with the history of epilepsy. This could be expected because data were collected in the clinical setting of an epilepsy surgery program. Surgery is preferred as the epilepsy burden (of which seizure frequency is an important factor) becomes subjectively unacceptable; surgical intervention might therefore be evaluated earlier in the disease course when patients suffer from seizures more frequently. Seizure frequency must be considered as possible confounder in this study. However, a Bonferroni correction was performed and our results regarding small world index remained significant, whereas the local clustering reached a p-value that was the same as the cut-off point for significance. We have found our results to be evidence of a robust correlation between epilepsy duration and these network characteristics. Apart from that, seizure frequency is reported as an extrapolation of the number of seizures patients suffered from at time of surgery, which does not truly represent the total number of seizures patients have had. Due to the retrospective nature of this study, we were not able to trace the absolute total number of seizures that patients had suffered from. Future research should point out whether the number of seizures prior to surgery is of influence on network characteristics.

Recently, a correlation has been shown between small world network topology and cognitive functioning [44]. Moreover, changed network characteristics in brain tumour patients may be responsible for cognitive decline [45]. Cognitive deficits are also an important quality of life limiting factor in temporal lobe epilepsy patients [46]. The correlation between disturbed functional networks and lesional epilepsy should therefore be subject of future studies.

### Conclusion

In the present study we have shown that there is both a decrease in connectivity of functional networks in the temporal lobe and that those same networks are more random in TLE patients having a long history of epilepsy. In particular we speculate that the functional neural networks of TLE patients become more random due to the influence of ongoing seizures on the structure of the network, irrespective of the presence of a structural lesion. Therefore our findings suggest that, as far as neural network functioning is concerned, surgical intervention might be more effective if performed earlier on in the course of TLE. Further investigations should address the course of epilepsy in TLE patients from the perspective of plasticity of network characteristics that accompany this process.

## Supporting Information

Figure S1 Epoch of ECoG data. Example of one epoch (8.19 seconds; sample rate 500 Hz) ECoG data, filtered in the broad frequency band 0.5–48 Hz. The intervals with low amplitude were interpreted as burst suppression periods when lasting more then 0.5 seconds.
(4.48 MB TIF)
