# Supplementary figures and images for: Long-Term Effects of Temporal Lobe Epilepsy on Local Neural Networks: A Graph Theoretical Analysis of Corticography Recordings
Source: PLoS One. 2009 Nov 26;4(11):e8081. doi: 10.1371/journal.pone.0008081 (PMC2778557; doi:10.1371/journal.pone.0008081)

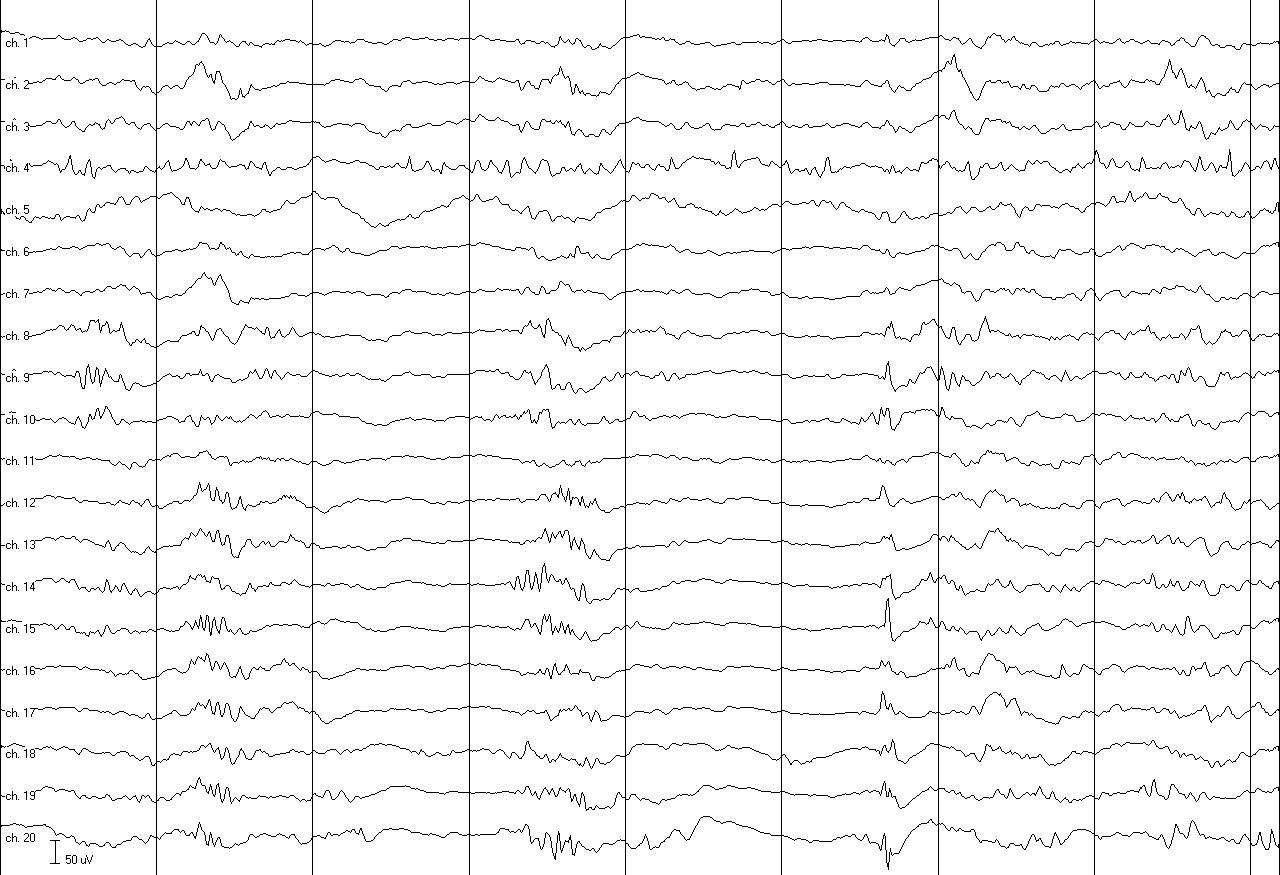

Supplement: Figure S1 — Epoch of ECoG data. Example of one epoch (8.19 seconds; sample rate 500 Hz) ECoG data, filtered in the broad frequency band 0.5–48 Hz. The intervals with low amplitude were interpreted as burst suppression periods when lasting more then 0.5 seconds. (4.48 MB TIF) [file pone.0008081.s001.tif]
